# Exploring the Most Effective Apical Seal for Contemporary Bioceramic and Conventional Endodontic Sealers Using Three Obturation Techniques

**DOI:** 10.3390/medicina59030567

**Published:** 2023-03-14

**Authors:** Hira Akhtar, Farah Naz, Arshad Hasan, Anum Tanwir, Danish Shahnawaz, Umair Wahid, Fariha Irfan, Muhammad Adeel Ahmed, Khalid H. Almadi, Mazen F. Alkahtany, Tariq Abduljabbar, Fahim Vohra

**Affiliations:** 1 Department of Operative Dentistry, Dow Dental College, Dow University of Health Sciences, Karachi 74200, Pakistan; hira.akhtar@duhs.edu.pk (H.A.); arshad.hasan@duhs.edu.pk (A.H.); 2 Department of Operative Dentistry, Dow International Dental College, Dow University of Health Sciences, Karachi 74200, Pakistan; farah.naz@duhs.edu.pk; 3 Department of Pediatric Dentistry, Baqai Medical University, Karachi 75340, Pakistan; anum.tanwir@gmail.com (A.T.); danishmsn@hotmail.com (D.S.); 4 Department of Operative Dentistry, Dr. Ishrat-ul-Ebad Institute of Oral Health Sciences, Dow University of Health Sciences, Karachi 74200, Pakistan; umair.wahid@duhs.edu.pk; 5 Department of Operative Dentistry, Hamdard University, Karachi 75210, Pakistan; fariha.irfan@hamdard.edu.pk; 6 Department of Restorative Dental Sciences, College of Dentistry, King Faisal University, Al-Ahsa 31982, Saudi Arabia; 7 Department of Restorative Dental Sciences, Division of Endodontics, College of Dentistry, King Saud University, Riyadh 11451, Saudi Arabia; kalmadi@ksu.edu.sa (K.H.A.); malkahtany@ksu.edu.sa (M.F.A.); 8 Department of Prosthetic Dental Sciences, College of Dentistry, King Saud University, Riyadh 11451, Saudi Arabia; tajabbar@ksu.edu.sa (T.A.); fvohra@ksu.edu.sa (F.V.)

**Keywords:** bioceramic sealer, calcium hydroxide, root canal obturation, Sealapex, Endoseal, Endosequence

## Abstract

*Background and Objective:* Despite a plethora of studies conducted to date, researchers continue to investigate the best sealer and obturation technique combinations. The aim of this study is to compare the apical seal provided by two bioceramic sealers (Endoseal and Endosequence) with that provided by a calcium hydroxide sealer (Sealapex), and to evaluate the effect of different obturation techniques (cold lateral condensation, continuous wave compaction and single cone) on the apical seal under a stereomicroscope. *Materials and Methods:* A total of 110 single-rooted mandibular premolar teeth were decoronated, cleaned and shaped using the Endosequence filing system to tip size 30/0.04 taper. Canals were irrigated with 5.25% NaOCl and 17% EDTA. The samples were randomly divided into 11 groups (9 experimental and 2 control groups) according to the designated sealer and technique. Samples were stored in an incubator for 7 days at 37 °C under 100% humidity. Samples were coated with nail varnish except for apical 2 mm and vertically placed in 0.2% rhodamine B dye solution for 48 h. Samples were split longitudinally and viewed under a stereomicroscope at 40× magnification. *Results:* Insignificant results were obtained between obturation techniques (*p* = 0.499) whereas statistically significant results were attained based on the type of endodontic sealer (*p* < 0.001). The overall lowest mean apical microleakage and best sealing ability was demonstrated by Sealapex (2.59 ± 1.20 mm) and amongst techniques by continuous wave compaction (3.90 ± 2.51 mm). *Conclusions:* Endosequence produced the best apical seal with the continuous wave compaction technique, whereas Endoseal did so with the bioceramic-coated single-cone technique. For the Sealapex sealer, the most effective apical seal was observed using cold lateral condensation. The quality and effectiveness of apical seal differed with the type of endodontic sealer and obturation technique used, and vice versa.

## 1. Introduction

Over the years, endodontology has evolved into an intricate discipline that has the capacity to successfully restore and retain infected and grossly carious teeth with a poor prognosis. Successful endodontic therapy aims to create a favorable environment for healing of periapical tissues via complete canal disinfection and formation of a three-dimensional apical seal. The quality of an apical seal is assessed by measuring the amount of apical microleakage that presents within and in-between the endodontic materials [1,2]. Obturation materials and techniques that facilitate increased penetration of sealer and gutta-percha (GP) into canal complexities are regarded as an effective mechanism in preventing microleakage and subsequent treatment failures [3].

Cold lateral condensation (CLC) is the gold standard of obturation methods and a benchmark against which other techniques are evaluated [4]. Despite this distinction, CLC is often criticized for producing a non-homogeneous obturation mass with distinct voids between master and accessory cones [5]. Hence, the ability of CLC to form an effective apical seal is unclear and requires exploration. Continuous wave compaction (CWC) and single cone (SC) techniques overcome the limitations of CLC by ensuring formation of a homogenous obturation mass, but the literature shows ambiguous data regarding the quality of apical seal achieved via these techniques [6]. Furthermore, the compatibility between CWC and new bioceramic sealers is questioned since heat generated during the procedure may alter sealer properties, thus affecting their sealing ability [7].

Martin et al. [8] agree that the durability of an apical seal is primarily dependent on the sealer, as it is the only anti-microbial obturation component offering resistance against bacteria and leakage [8]. Sealapex (Kerrdental, Brea, CA, USA) is a conventional calcium hydroxide sealer with a long and successful history of clinical use. It has antimicrobial activity, stimulates periapical tissue healing and promotes hard tissue formation [9]. In light of these advantages, Sealapex is commonly used in endodontics worldwide. The solubility of Sealapex has entailed a long-standing debate questioning the material’s ability to provide a durable seal. These concerns stem from its mechanism of action, which requires the material to undergo ionic disassociation into calcium and hydroxyl ions [9]. This is against the doctrine of Grossman’s ideal sealer properties, which insists that the sealer should remain insoluble in the presence of fluids [9].

Contemporary bioceramic sealers were designed to retain the desirable properties of conventional sealers while overcoming their limitations of solubility and shrinkage [10]. MTA has become the material of choice for many endodontic procedures. The material’s ability to set in the presence of moisture has granted it an unparalleled distinction over previous generations of sealer. Previous literature has repeatedly reported that MTA lacks the physio-chemical properties for an endodontic sealer [10]. Therefore, tireless efforts have been made to modify MTA, making it suitable for endodontic use. Endoseal MTA (Maruchi, Wonju, Korea) is a pre-mixed, injectable sealer intended for direct canal application [10]. It has been proposed to overcome difficulty of manipulation by performing single-cone obturation aided with ultrasonic power in order to obtain better root filling with fewer voids [11].

Endosequence (Brasseler, Savannah, GA, USA) is a new bioceramic sealer (BC) specifically designed to overcome the disadvantages associated with MTA sealers, such as discoloration and difficult manipulation [8]. The hydrophilic nature and easy manipulation of the material allow it to set in moisture, remain dimensionally stable and undergo slight expansion during setting. Moreover, it becomes hard and insoluble when completely set, ensuring a long-term seal [12]. It has been suggested that using BC-coated GP can aid in attaining a gap-free obturation, further improving the seal and preventing apical microleakage. However, clinical evidence supporting this claim is lacking [12,13]. Thus, the sealing ability of BC sealers over conventional sealers is undetermined and yet to be explored.

Today, researchers continue to investigate the best combinations of sealer and obturation technique. This was chosen as the theme of this study as it remains a topic of interest in the current literature. It is a pressing requirement that we determine the most effective sealer–obturation technique combination that will produce the best apical seal. This study aimed to compare the apical seal provided by two bioceramic sealers with that provided by a calcium hydroxide sealer, and to evaluate the effect of different obturation techniques on the apical seal under a stereomicroscope. This research will help clinicians to better understand the different obturation techniques and endodontic sealers currently available, the effect that these two entities have on each other and the role they play in achieving an effective apical seal.

## 2. Materials and Methods

### 2.1. Sample Size Calculation

Sample size was calculated using an Openepi calculator. The mean difference between two sealers was taken as 0.42 (Sealer 1: mean ± SD = 1.22 ± 0.53 and Sealer 2: mean ± SD = 0.8 ± 0.61) [14] with 80% power of test and 95% confidence interval. The total sample size calculated for two groups was 60, i.e., 30 cases in each sealer group.

In this study, each sealer group was further divided into 3 subgroups (on the basis of obturation technique). The sample size for each sealer group (*n* = 30) was equally distributed for each subgroup (*n* = 10). A total sample size of 110 teeth was selected for the study.

### 2.2. Sample Selection and Preparation

This experimental in vitro study was approved by the Institutional Ethics Committee (IRB-942/DUHS/Approval/2019/163). A total of 110 sound mandibular premolars with mature apices, straight roots and a single canal system, which were extracted due to orthodontic or periodontal reasons, were selected for the study. Following extraction, teeth were ultrasonically cleaned of all debris and stored in 0.1% thymol solution until further procedure.

Preparation of all samples was performed by the principal investigator. To prevent dehydration, samples were held in a moistened piece of gauze. Crowns were sectioned using a diamond disk with a water coolant attached in a slow-speed handpiece (Nakanishi, Japan) to obtain a standardized root length of 15 mm ± 1 mm. All samples showed a visible canal orifice through which #10 K-file (MANI, Japan) was inserted to check patency. Working length was determined by inserting a #15 K-file and subtracting 1 mm from the length when the file tip was visible at the apex. After a reproducible guide path was established, canal preparation was performed using the crown-down technique with the Endosequence filing system (Brasseler, USA) at 600 rpm and 2.0 Ncm torque settings. Complete cleaning and shaping were performed to tip size 30/0.04 taper. Irrigation was performed with 5.25% NaOCl. After preparation was complete, samples were irrigated with 5 mL of 17% EDTA for 1 min to remove the smear layer. Finally, the canals were flushed with 2 mL of distilled water for 1 min and dried with paper points (Brasseler, USA).

### 2.3. Obturation of Samples

The samples were randomly divided into 11 groups (9 experimental and 2 controls) with an equal sample (*n* = 10) in each group.

Group 1: Sealapex with CLC technique

Group 2: Sealapex with CWC technique using Elements obturation unit (EOU)

Group 3: Sealapex with SC technique using bioceramic (BC)-coated GP

Group 4: Endosequence with CLC technique

Group 5: Endosequence with CWC technique using EOU

Group 6: Endosequence with SC technique using BC-coated GP

Group 7: Endoseal with CLC technique

Group 8: Endoseal with CWC technique using EOU

Group 9: Endoseal with SC technique using BC-coated GP

Group 10: Negative control

Group 11: Positive control

### 2.4. Sealer Application

Endosequence and Endoseal are available as premixed pastes whereas Sealapex is dispensed as a two-paste system. Sealapex was mixed as per the manufacturer’s instructions by dispensing equal lengths (5 mm) of base and catalyst pastes onto a mixing pad and homogeneously mixing using a cement spatula. A #25 lentulospiral (MANI, Japan) was attached to a slow-speed handpiece (Nakanishi, Japan) and used for sealer placement. The 5 mm tip of the lentulospiral was dipped twice in sealer to obtain an even coating. All lentulospiral sealer applications were performed 2 mm short of the working length to prevent extrusion.

### 2.5. CLC Obturation

After confirming tug-back and designated sealer application in the canal, the apical 2 mm of the master cone was evenly coated with sealer and inserted up to the working length. Finger spreaders #25 and #20 were sequentially introduced to create space and accessory GPs were inserted. The procedure was repeated until the #20 spreader could not penetrate past the coronal one-third of the canal. Excess GP was seared off and vertical compaction of coronal GP was carried out with a condenser.

### 2.6. CWC Obturation Using EOU

All obturations were carried out as per the manufacturer’s instructions. The apical tip of the master cone was cut 1 mm short of the working length and tug-back was confirmed. A 0.06 taper plugger was trial fitted into the canal space. An apical binding point 3 mm short of the apex was noted and the rubber stopper was adjusted accordingly. After lentulospiral sealer application was completed, the apical 2 mm of GP was evenly coated and inserted into the canal. The plugger was activated (200 °C) and gently inserted into the canal through the GP mass until it reached the binding point. The plugger was de-activated and cooling (condensation) pressure was maintained for 10 s. To remove the plugger from the solidified GP mass, heat was applied to create a separation burst [15]. The remaining canal space was backfilled using an extruder handpiece with a 23 gauge needle, and vertical condensation of coronal GP was performed.

### 2.7. SC Obturation with BC-Coated GP

After confirming tug-back and designated sealer application, the apical 2 mm of the master cone was evenly coated and inserted up to the working length. Excess GP was seared off and vertical compaction of coronal GP was completed.

Following obturation, all access cavities were sealed with restorative-type GIC (3 M, ESPE). All control group samples were prepared in a similar manner as described above. For positive control, the samples were left unfilled, whereas negative control samples were obturated with Sealapex and the CLC technique. To promote complete sealer setting, all samples were stored in an incubator (Binder GmbH) at 37 °C under 100% humidity for 1 week.

### 2.8. Stereomicroscopic Analysis of Dye Penetration

All experimental group samples were evenly coated with two layers of colored nail polish, with the exception of their apical 2 mm. The negative controls were entirely coated with nail polish (including their apical foramen), whereas positive control samples did not receive any nail polish application. Following complete drying of nail polish, the samples were vertically placed in 0.2% rhodamine B (Avonchem, Macclesfield, UK) dye solution for 48 h. They were then rinsed under running tap water and air dried.

Nail polish and dye was scraped off the samples using a #15 surgical blade to prevent dye introduction onto the inner root surface at the time of sectioning. The roots were split longitudinally parallel to the long axis of the tooth. To ensure that the sectioning was performed through the center of the root, 2 mm deep longitudinal grooves were made in the middle of the buccal and lingual root surfaces using a diamond disk in a slow-speed handpiece, with caution taken not to invade and damage the canal space. The teeth were then fractured longitudinally with a double-tapered chisel and mallet, preserving the entire obturation space [1].

A random sequence of the samples was generated via computer software (MS Excel) and allocated to subgroups. The samples were viewed under a stereomicroscope (Olympus VM-ILA-2) at 40× magnification. The images were captured and uploaded to IMAGE J software (National Institute of Health, Bethesda, Maryland, USA) and blind analyzed by two examiners. For assessment of apical microleakage, investigators measured the linear depth of dye penetration by recording the maximum distance traveled by the dye from the root apex up to its coronal extent at the sealer–dentinal wall interface. All measurements were calculated in millimeters (mm).

### 2.9. Statistical Analysis

SPSS version 23 (SPSS Inc., Chicago, IL, USA) was used for data analysis. Intra-class correlation was used to determine intra- and inter-observer reliability. For determination of intra-observer reliability, 60 sample images were randomly selected and re-evaluated after 2 weeks by the same examiners.

The Shapiro–Wilk test showed non-normal data distribution. Kruskal–Wallis testing was used for mean comparison of all sealers and techniques. A series of Mann–Whitney tests were applied for multiple pairwise comparisons. Significance for the purposes of post hoc tests was adjusted using the Bonferroni method; a *p*-value < 0.001 was considered significant.

The Bonferroni adjustment method was used to counteract the statistical type I error in the study resulting from performing multiple Mann–Whitney comparisons.

## 3. Results

A total of 110 mandibular premolar teeth were stereomicroscopically assessed to evaluate the apical sealing ability of three endodontic sealers using three different obturation techniques. Excellent intra-observer reliability was recorded for both examiners (Examiner 1: 0.93; Examiner 2: 0.988). Inter-observer reliability was measured as 0.992, signifying an excellent correlation between the two examiners. All specimens in the positive control demonstrated maximum levels of dye penetration whilst no dye penetration was observed in negative control samples.

With regards to sealing ability, insignificant results were obtained between obturation techniques (*p* = 0.499) whereas statistically significant results were attained based on the type of endodontic sealer (*p* < 0.001). The overall lowest mean apical microleakage and best sealing ability amongst sealers was demonstrated by Sealapex (2.59 ± 1.20 mm) and amongst techniques by continuous wave compaction (3.90 ± 2.51 mm) whereas the highest dye penetration was observed for Endoseal sealer (7.08 ± 0.67 mm) and the single-cone technique (4.49 ± 1.91 mm). In terms of sealer–technique combinations, minimum dye penetration was observed in Sealapex with CLC (Group 1: 1.81 ± 0.49 mm) and the maximum value was recorded for Endoseal with CLC (Group 7: 7.48 ± 0.30 mm) (Table 1).

A pairwise sealer comparison showed an insignificant difference between the sealing abilities of Sealapex and Endosequence (*p* = 0.348) whereas statistically significant results were observed when Endoseal was compared with the Sealapex and Endosequence sealers (*p* < 0.001) (Table 2).

Multiple pairwise comparisons between all sealer–technique combination groups were evaluated to compare all obturations. Endoseal groups (groups 7, 8 and 9) showed the most significant differences when compared to other sealer–technique combinations (Table 3).

## 4. Discussion

It is a universally accepted view that formation of a three-dimensional apical seal is an essential part of successful endodontic treatment. In light of this endodontic goal, the purpose of the present study was to perform a comparative apical seal analysis between contemporary and conventional sealers on the basis of three different obturation techniques. Interestingly, all three endodontic sealers produced their most effective apical seal with different obturation techniques. Furthermore, we found that the apical seal has a significant association with the endodontic sealer employed, but non-significant association with the obturation technique used.

Over 80% of microleakage studies have opted for dye penetration as the assessment method of choice [1,2]. In the present study, linear dye penetration was measured in millimeters to yield a true, precise and quantitative microleakage assessment. Conventional methylene blue (MB) studies infer false microleakage results. Previous studies have proven MB to be unstable and undergo hydrolysis into a colorless compound called thionine when in the presence of endodontic materials (calcium silicates and calcium hydroxide sealers) that produce an alkaline environment during setting [16,17]. Recent literature has advocated for the use of fluorescent dyes over MB when evaluating leakage of alkaline materials [18]. Keeping in accordance with current literature, we chose fluorescent rhodamine B dye for assessment. Excellent intra- and inter- observer reliabilities observed in the study signify that rhodamine B dye penetration possesses distinct and sharp visualization properties, thus proving to be a reliable and reproducible method for assessment of apical sealing ability of alkaline endodontic sealers.

In the present study, amongst the sealer groups tested, Sealapex demonstrated the lowest apical microleakage followed by Endosequence; highest values were observed for Endoseal. The better sealing ability of Sealapex can be credited to the materials’ property of volumetric expansion. On the contrary, it is argued in the literature that, since Sealapex undergoes volumetric expansion via water absorption to attain an initial acceptable seal, this paradoxically causes the material to become soluble, thus deteriorating its apical seal over time [1,2]. The solubility of Sealapex has always been a source of contention and criticism within the dental community. Colombo et al. evaluated the physio-chemical properties of different endodontic sealers; they concluded that Sealapex demonstrated a solubility of <3%, which fulfils ISO and ANSI/ADA specifications for an endodontic sealer. Moreover, they reported higher solubility values for MTA Fillapex in comparison with Sealapex [19]. Another study by Altan et al. evaluated the short- and long-term seal of MTA Fillapex, AH Plus and Sealapex at 24 h and 6 month durations. They reported a negative correlation between time and sealing ability of Sealapex. Their study concluded that MTA Fillapex provided the best short-term seal at 24 h whereas the sealing ability of Sealapex significantly improved with time, producing the best seal at 6 months [20].

In the current study, amongst the calcium silicate sealers tested, Endosequence produced better results than Endoseal. Similar outcomes were reported by Ballullaya et al.; they performed stereomicroscopic analysis of six sealers and reported better sealing ability of Endosequence and Sealapex in comparison to MTA Plus [21]. Similar observations to those of the present study were reported by Baruah et al. after comparing Endosequence and MTA Fillapex. They concluded that its small particle size, hydrophilic nature and low contact angle offer Endosequence the advantage of spreading and flowing easily into narrow canal spaces, which accounts for its better sealing ability [22]. Moreover, the ability of Endosequence to bond with dentin and BC-coated GP can be credited for its better obturation and seal, as observed in the present study. This observation is supported by previous research [23,24].

In our study, all three Endoseal technique groups yielded statistically significant differences (*p* < 0.001) when compared to other sealer–technique combinations. According to the manufacturer, Endoseal creates an excellent seal; however, recent studies have shown conflicting data regarding this claim [25,26]. One of the reasons for recording high Endoseal microleakage in the present study could be the lack of use of ultrasonic (US) activation during obturation. The literature encourages cautious use of US vibration at 28–36 Hz to remove entrapped air bubbles in order to enhance sealer flowability and help minimize obturation flaws [27]. Previous studies have reported that US activation produced better homogenous Endoseal obturations with increased dentinal tubular sealer penetration [24,27].

A pattern of observations similar to those of the current study has been seen in previous research, where MTA-based sealers have shown higher microleakage values than those of conventional materials after 7 days [28,29]. Similar to our findings, other authors speculate that high initial solubility of Endoseal may be responsible for the greater microleakage values observed. Previous studies have demonstrated that Endoseal exhibits high initial fluid intake, solubility and porosity, which reduce over a period of 28 days [30]. These findings negate the manufacturer’s claim of low material solubility (0.7%) [30] and require further research. Another explanation for recording high microleakage could be lack of bonding between Endoseal and GP, and inferior bond strength between MTA sealers and dentin. These observations are in accordance with previous studies by Silva et al. [31] and Atom et al. [3]. The presence of scientific evidence of low bond strength between MTA sealers and dentin contradicts the manufacturer’s claim and requires further exploration. The overall inferior bonding of Endoseal to dentin, coupled with a lack of bonding between the sealer and GP, and difficulty of manipulation, could result in formation of voids or improper sealer coating of canal walls. These could be the reasons for high microleakage values observed in Endoseal obturation groups in the current study.

The present study also aimed to evaluate the effect of three different obturation techniques on the apical sealing ability of conventional and contemporary sealers. The results showed insignificant differences between the three obturation methods (*p* = 0.449). Similar to our findings, previous research has concluded that the degree of sealer penetration seems to be independent of the type of obturation technique used [18,32]. In terms of the small differences, we observed that the CWC technique provided the lowest microleakage values whereas the highest microleakage levels were recorded with the SC technique using BC-coated GP. Previous studies have reported that warm compaction techniques adversely affect the sealing ability of calcium silicate sealers as heat negatively affects flow, setting time and residual moisture content in canals and dentinal tubules [7,33,34,35]. Contrary to these findings, other authors have reported favorable results when using the CWC technique and calcium silicate sealers. Tanompetsanga P. and Tungsawat P. assessed Endosequence with SC and CWC techniques and reported significantly higher microleakage with the SC technique [36]. The better sealing ability achieved via the CWC technique in the present study can be attributed to the application of vertical compaction pressure. That encourages greater penetration of GP and sealer into canal complexities, which, in turn, may be responsible for improving apical seals.

In sum, this is the first study that compares the apical sealing ability of contemporary calcium silicate sealers with a conventional calcium hydroxide sealer on the basis of three different obturation techniques. There were a few limitations to the study: for example, non-invasive assessment methods such as micro CT could not be employed due to lack of availability and limited budget; the effect of canal curvature was not assessed; and small subgroup sample sizes were used. These areas should be improved in future research.

## 5. Conclusions

Both contemporary and conventional endodontic sealers exhibited varied apical sealing ability when used with different obturation techniques. Interestingly, in the present study, amongst the sealer groups tested, Sealapex demonstrated the lowest apical microleakage, followed by Endosequence, whereas the highest values were observed for Endoseal.

## Figures and Tables

**Table 1:** Means and standard deviations of apical microleakage of all endodontic sealers and techniques.

Sealers	Techniques				*p*-Value *
	**Cold Lateral Condensation**	**Continuous Wave Compaction**	**Single Cone**	**Sealers** **Mean ± SD**	<0.001
	Mean ± SD *	**Mean ± SD**	**Mean ± SD**		
**Sealapex**	1.81 ± 0.49	2.63± 1.03	3.32 ± 1.43	2.59 ± 1.20	
**Endosequence**	3.50 ± 1.86	1.93 ± 1.10	3.55 ± 1.20	2.99 ± 1.58	
**Endoseal**	7.48 ± 0.30	7.16 ± 0.29	6.60 ± 0.92	7.08 ± 0.67	
**Techniques** **Mean ± SD**	4.26 ± 2.65	3.90 ± 2.51	4.49 ± 1.91		
***p*** -Value **	0.499				

*p*-values calculated using Kruskal–Wallis test. *p*-value < 0.05 was considered significant. * Standard deviation * *p*-value for endodontic sealers and ** *p*-value for obturation techniques.

**Table 2:** Pairwise comparison of different endodontic sealers.

Sealers	Absolute Mean Differences	*p*-Value *
Sealapex vs. Endosequence	0.408	0.348
Sealapex vs. Endoseal	4.493	<0.001
Endosequence vs. Endoseal	4.085	<0.001

*p*-value calculated using Mann–Whitney test. * *p*-value < 0.05 was considered significant.

**Table 3:** Multiple comparisons between all sealer–technique groups.

Groups	Mean Difference	95% Confidence Interval (Lower to Upper Bound)	*p*-Value *
**Group 1 vs. 2**	−0.820	−2.419 to 0.779	0.008
**Group 1 vs. 3**	−1.508	−3.107 to 0.091	0.005
**Group 1 vs. 4**	−1.691	−3.290 to −0.092	0.006
**Group 1 vs. 5**	−0.124	−1.723 to 1.475	0.705
**Group 1 vs. 6**	−1.737	−3.336 to −0.138	0.002
**Group 1 vs. 7**	−5.670	−7.269 to −4.071	<0.001
**Group 1 vs. 8**	−5.350	−6.949 to −3.751	<0.001
**Group 1 vs. 9**	−4.786	−6.385 to −3.187	<0.001
**Group 2 vs. 3**	−0.688	−2.287 to 0.911	0.257
**Group 2 vs. 4**	−0.871	−2.470 to 0.728	0.290
**Group 2 vs. 5**	0.696	−0.903 to 2.295	0.590
**Group 2 vs. 6**	−0.917	−2.516 to 0.682	0.700
**Group 2 vs. 7**	−4.850	−6.449 to −3.251	<0.001
**Group 2 vs. 8**	−4.530	−6.129 to −2.931	<0.001
**Group 2 vs. 9**	−3.966	−5.565 to −2.367	<0.001
**Group 3 vs. 4**	−0.183	−1.782 to 1.416	1.000
**Group 3 vs. 5**	1.384	−0.215 to 2.983	0.028
**Group 3 vs. 6**	−0.229	−1.828 to 1.370	0.520
**Group 3 vs. 7**	−4.162	−5.761 to −2.563	<0.001
**Group 3 vs. 8**	−3.842	−5.441 to −2.243	<0.001
**Group 3 vs 9**	−3.278	−4.877 to −1.679	<0.001
**Group 4 vs. 5**	1.567	−0.032 to 3.166	0.026
**Group 4 vs. 6**	−0.046	−1.645 to 1.553	0.597
**Group 4 vs. 7**	−3.979	−5.578 to −2.380	<0.001
**Group 4 vs. 8**	−3.659	−5.258 to −2.060	<0.001
**Group 4 vs. 9**	−3.095	−4.694 to −1.496	0.0014
**Group 5 vs. 6**	−1.613	−3.212 to −0.014	0.004
**Group 5 vs. 7**	−5.546	−7.145 to −3.947	<0.001
**Group 5 vs. 8**	−5.226	−6.825 to −3.627	<0.001
**Group 5 vs. 9**	−4.662	−6.261 to −3.063	<0.001
**Group 6 vs. 7**	−3.933	−5.532 to −2.334	<0.001
**Group 6 vs. 8**	−3.613	−5.212 to −2.014	<0.001
**Group 6 vs. 9**	−3.049	−4.648 to −1.450	<0.001
**Group 7 vs. 8**	0.320	−1.279 to 1.919	0.019
**Group 7 vs. 9**	0.884	−0.715 to 2.483	0.002
**Group 8 vs. 9**	0.564	−1.035 to 2.163	0.041

*p*-value calculated using Mann–Whitney test and post hoc Bonferroni test. * *p*-value < 0.001 was considered significant.

## Data Availability

Not applicable.
